# Preparation and Properties of Alkali Activated Metakaolin-Based Geopolymer

**DOI:** 10.3390/ma9090767

**Published:** 2016-09-08

**Authors:** Liang Chen, Zaiqin Wang, Yuanyi Wang, Jing Feng

**Affiliations:** 1 Materials and Structural Department, Changjiang River Scientific Research Institute, Wuhan 430010, China; wangzq@mail.crsri.cn (Z.W.); wangyuanyi@mail.crsri.cn (Y.W.); fengjing@mail.crsri.cn (J.F.); 2 Collaborative Innovation Center for Geo-Hazards and Eco-Environment in Three Gorges Area, Yichang 443002, China

**Keywords:** alkali activated, geopolymer, metakaolin, curing condition, thermal insulation

## Abstract

The effective activation and utilization of metakaolin as an alkali activated geopolymer precursor and its use in concrete surface protection is of great interest. In this paper, the formula of alkali activated metakaolin-based geopolymers was studied using an orthogonal experimental design. It was found that the optimal geopolymer was prepared with metakaolin, sodium hydroxide, sodium silicate and water, with the molar ratio of SiO_2_:Al_2_O_3_:Na_2_O:NaOH:H_2_O being 3.4:1.1:0.5:1.0:11.8. X-ray diffraction (XRD) and Fourier transform infrared spectroscopy (FT-IR) were adopted to investigate the influence of curing conditions on the mechanical properties and microstructures of the geopolymers. The best curing condition was 60 °C for 168 h, and this alkali activated metakaolin-based geopolymer showed the highest compression strength at 52.26 MPa. In addition, hollow micro-sphere glass beads were mixed with metakaolin particles to improve the thermal insulation properties of the alkali activated metakaolin-based geopolymer. These results suggest that a suitable volume ratio of metakaolin to hollow micro-sphere glass beads in alkali activated metakaolin-based geopolymers was 6:1, which achieved a thermal conductivity of 0.37 W/mK and compressive strength of 50 MPa. By adjusting to a milder curing condition, as-prepared alkali activated metakaolin-based geopolymers could find widespread applications in concrete thermal protection.

## 1. Introduction

Alkali activated geopolymers have attracted increasing attention in recent years for their potential uses as construction materials and environmentally cementitious binders that would partly replace ordinary Portland cement (OPC) [1,2]. Studies have shown that geopolymers have excellent mechanical properties [3], such as high early strength and low shrinkage [4,5]. In addition, geopolymers also can be used as construction panels and fire resistant materials [6] for their characteristic light weight and thermal insulation properties [7,8,9]. Geopolymers can be produced from many materials, even waste materials. Metakaolin, fly ash, kaolin, and slag are the typical raw materials for the preparation of geopolymer composites [10].

Metakaolin is a de-hydroxylated form of the clay mineral kaolinite with 4-, 5-, and 6-coordinated aluminum ions in alumina polyhedron sheet structures [11]. Metakaolin is also a valuable admixture with many excellent advantages, including porosity, high specific area, good absorbability and strong coordinative bonds when stimulated [12]. The initial reaction process and performance of alkali activated metakaolin-based geopolymer is not only influenced by the chemical composition [13], dosage and concentration of raw materials [14,15,16] but also affected by the curing conditions during the early-age polymerization process [17,18].

In addition to the above characteristics, one of the applications of geopolymers is its use as a protective coating material. As a type of inorganic polymeric silicate material with three-dimensional network structure based on covalent and ionic bonds, geopolymers have the advantages of strength, excellent mechanical properties and adjustable setting time. The excellent acid and alkali corrosion resistance and high temperature resistance properties of geopolymers are absent in most common organic concrete protective materials. Therefore, alkali activated metakaolin-based geopolymers may also have potential application prospects in concrete surface protection [19,20].

In this paper, an alkali activated metakaolin-based geopolymer was prepared with metakaolin, sodium hydroxide, sodium silicate and water. The formulation of raw materials for the geopolymer was studied using an orthogonal experimental design. The influence of curing temperature and time on the mechanical properties and microstructures of alkali activated metakaolin-based geopolymers were investigated by X-ray diffraction (XRD) and Fourier transform infrared spectroscopy (FT-IR). In addition, hollow micro-sphere glass beads (HMGB) were mixed with metakaolin particles to improve the thermal insulation property of the alkali activated metakaolin-based geopolymer. The internal morphologies, thermal conductivity and compressive strength of different HMGB modified geopolymers were studied. We also explored the interfacial properties and durability of prepared glass bead modified geopolymers as a coating on concrete. The results suggest that by adjusting the curing condition to a milder condition, as-prepared alkali activated metakaolin-based geopolymers could find widespread applications in concrete thermal protection.

## 2. Experimental Methods

### 2.1. Characterization

The composition of metakaolin powder was determined by X-ray Fluorescence (Axios advanced, PANalytical B.V., Almelo, The Netherlands) after tabletting treatments. The micro-morphologies of the alkali activated geopolymer were analyzed with scanning electron microscopy (SEM, JSM-6610LA, JEOL Ltd., Tokyo, Japan). The compressive strength of the samples (cube mold, 20 mm × 20 mm × 20 mm) after curing were measured on a universal force tester (AG-IC 100KN, Shimadzu, Kyoto, Japan) at a sliding velocity of 1 mm/min. In the compressive strength measurements, three parallel samples were tested for each specimen. FT-IR measurements were performed by a Bruker TENSOR27 spectrometer (Bruker Optik GmbH, Ettlingen, Germany) after the cured geopolymers were ground into powder. The X-ray diffraction (XRD) patterns were also measured on geopolymer powder over the scattering 2θ angle range 10°–90° using CuKα radiation (λkα_1_ = 1.54056 Å, λkα_2_ = 1.54439 Å) with a step-size of 0.0194° at 25 °C (D8 Advance, Bruker). The adhesive strength was tested by PosiTest pull-off tester (DeFelskoAT-A, Ogdensburg, NY, USA) according to ISO 4624/16276-1. The thermal conductivity of 10 cm thick alkali activated geopolymer coating was determined by a thermal conductivity measuring apparatus (QTM-500, Kyoto Electronics Manufacturing Company, Kyoto, Japan). Artificial weathering aging tests were performed according to ISO 4892-2 with a Xenon Test Chamber (Q-SUN Xe-1-S, Q-Lab Corporation, Westlake, OH, USA). The irradiance was set at 0.51 W/m^2^ with 340 nm wavelength. The inside temperature of the chamber remained at 50 ± 5 °C. Every 2 h, 102 min of radiation and 18 min of water spay were alternately carried out on the samples (100 mm × 100 mm × 10 mm). After 1000 cycles (2000 h), the samples were taken out of the chamber for further observation and evaluation of appearance.

### 2.2. Materials

Highly reactive metakaolin (trade name METAMAX) was purchased from BASF SE (Ludwigshafen, Germany). The chemical composition results of the metakaolin determined by X-ray fluorescence (XRF) is listed in Table 1. Industrial sodium silicate solution (Na_2_O·nSiO_2_, *n* ≈ 2.4, mass fraction W_(__Na2O__+SiO2__)_% ≈ 36%) was obtained from Tongxiang Sunny Sodium Silicate Plant (Wuhan, China). Sodium hydroxide was purchased from SINOPHARM Chemical Reagent Co., Ltd. (Beijing, China). HMGB were purchased from 3M Company (trade name K1 and VS-5500, St. Paul, MN, USA). The main performance parameters of HMGB K1 and HMGB VS-5500 are listed in Table 2. Compared with HMGB K1, HMGB VS-5500 possessed a much higher compressive strength as well as a smaller diameter with more narrow size distribution. All of the above reagents were used as received without further modification.

### 2.3. Sample Preparation

The basic formula of the alkali activated metakaolin-based geopolymer consisted of metakaolin, sodium hydroxide, sodium silicate, water and hollow micro-sphere glass beads (if needed). To explore the impact on the compressive strength of the geopolymer, we chose four components (metakaolin, sodium hydroxide, sodium silicate and water) as the three-level factors in an orthogonal experimental design demonstrated in Table 3. The samples were prepared by the following procedure. First, sodium hydroxide was dissolved in water to prepare a strong alkali solution. Sodium silicate solution was then added to the alkali solution and stirred for 5 min until the alkali solution cooled down to room temperature. After that, metakaolin or a mixture of metakaolin and HMGB were sieved into the alkali solution and stirred well to form a slurry paste. Finally, alkali activated metakaolin-based geopolymer was obtained after the curing procedure at different curing temperatures (20 °C, 40 °C, 60 °C, 80 °C and 100 °C) and curing times (24 h, 72 h and 168 h) with an initial relative humidity of 50% ± 5% during the first 12 h.

## 3. Results and Discussion

### 3.1. Formula of Alkali Activated Metakaolin-Based Geopolymer

Multiple factors determine the properties, especially the compressive strength, of alkali activated geopolymers, such as the type and concentration of alkaline-activator, the proportion of raw materials, curing conditions, and so on. In this paper, we discuss the impact of four components on the mechanical properties of geopolymer. As demonstrated in Table 4, slurries of alkali activated metakaolin-based geopolymer were prepared according to the ratios of an L9(3^4^) orthogonal arrays. Here, L9(3^4^) represents an orthogonal test designed for four factors with three levels for each factor, as listed in Table 3. Specimens #1 through #9 of the prepared slurries were cured at 20 °C for seven days for compressive strength tests. In the compressive strength tests, three specimens were tested of each sample, for which the average compressive strength and standard deviation data are listed in Table 4. Based on the range analysis of the orthogonal experiment in Table 4, it is suggested that the content of the alkaline-activator, NaOH, had the most important impact on the compressive strength, followed by the content of metakaolin, water and sodium silicate. Furthermore, it can be concluded from the range analysis that the optimal mass ratio of alkali activated metakaolin-based geopolymer was sample #5, with the highest compressive strength of 37.4 MPa. Calculated from the chemical composition of raw materials, the oxide molar ratio of SiO_2_:Al_2_O_3_:Na_2_O:H_2_O in sample #5 was 3.4:1.1:1.0:11.8. This ratio of sample preparation was adopted in further samples.

### 3.2. Influence of Curing Temperature and Time on the Properties of Alkali Activated Metakaolin-Based Geopolymer

The performance of alkali activated metakaolin-based geopolymers are not only influenced by the chemical composition, dosage and concentration of raw materials but also by the curing conditions during the early-age polymerization process. In this paper, the influence of curing temperature and time on the properties of alkali activated metakaolin-based geopolymers will be discussed.

#### 3.2.1. Influence of Curing Temperature and Time on the Compressive Strength of Alkali Activated Metakaolin-Based Geopolymers

**Figure 1:**
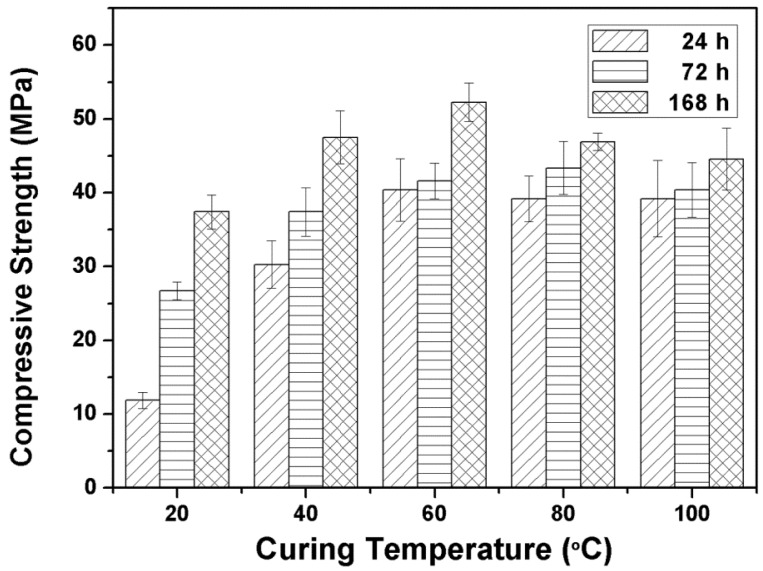
Effects of curing temperature and time on the compressive strength of alkali activated metakaolin-based geopolymer.

As demonstrated in Figure 1, the relationship of compressive strength of alkali activated metakaolin-based geopolymers with curing temperature and time was studied at 20 °C, 40 °C, 60 °C, 80 °C and 100 °C for 24 to 168 h. The samples were prepared using the weight ratios referred to as sample #5 in Section 3.1. After 24 h of thermal curing, all geopolymers had solidified from metakaolin paste to form rigid structures. However, it was found that the compressive strength was only 11.88 MPa after 24 h of curing at 20 °C. This value increased to 37.41 MPa after curing for 168 h, which indicates that the polymerization rate of the geopolymer slurry was quite slow at relatively low temperatures [21]. The results also suggest that the best curing condition was 60 °C for 168 h, where the alkali activated metakaolin-based geopolymer showed the highest compressive strength at 52.26 MPa.

The increased curing temperature facilitates the dissolution of amorphous structures in metakaolin particles and promotes the formation and polymerization of the alkali-activation precursors, such as OSi(OH)_3_^−^, Al(OH)_4_^−^ and (OH)_3_^−^Si-O-Al-(OH)_3_ coordination structures [22]. In addition, raising the curing temperature is also advantageous to the exclusion of water in the reaction system, which accelerates the growth of the gel phase [21]. Although the metakaolin paste could not solidify in the initial 12 h at 20 °C, under higher temperature conditions, the metakaolin paste solidified within a short time to form rigid structures with great strength. In summary, the compressive strength of samples cured at a higher temperature are greater than those cured at a low temperature.

On the other hand, the dissolution of the amorphous structure of metakaolin particles becomes too fast at higher curing temperature conditions, e.g., 80 °C and 100 °C. Stimulated by the alkali activator, polymerization between Al atoms in metakaolin and Si monomers or oligomers occurs rapidly, and metakaolin particles become sealed by a layer of geopolymer, preventing contact with the alkali activator [21]. In addition, when the curing temperature was high, the samples experienced a substantial loss of moisture, which is required for the geopolymer reaction to develop strength. We found the same trend reported in the literature, whereby increasing the curing temperature beyond 60 °C does not substantially increase the compressive strength after curing for 168 h [23,24].

#### 3.2.2. Influence of Curing Temperature and Time on the Microstructure of Alkali Activated Metakaolin-Based Geopolymer

**Figure 2:**
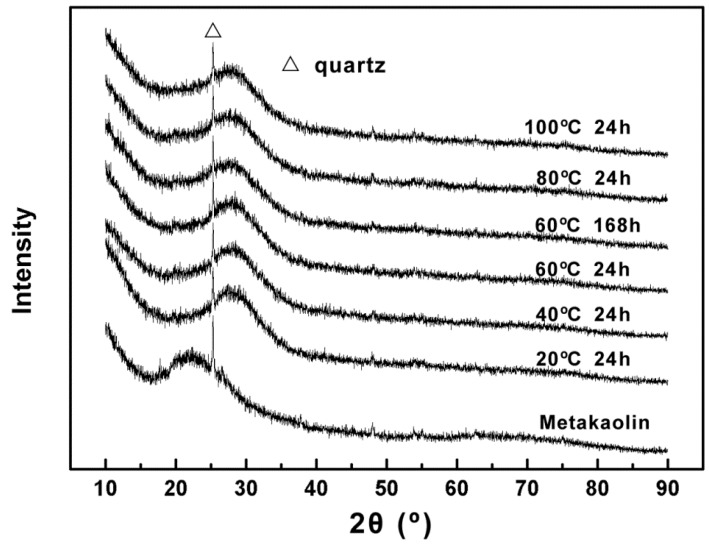
X-ray diffractograms of metakaolin and geopolymers cured at different temperatures and times.

Figure 2 shows the X-ray diffractograms of raw material metakaolin and geopolymers cured at different temperatures and times. In the XRD pattern of metakaolin, a wide dispersion peak located between 18° and 25° was attributed to the amorphous structure of metakaolin [25]. In addition to quartz crystals, the microstructure of metakaolin was mainly hypocrystalline and amorphous. After geopolymerization, all XRD patterns showed the typical amorphous structure of metakaolin geopolymer with a wide diffraction hump in the range of 20° to 35° (2θ), regardless of the curing condition differences. As assumed by Palomo et al. and Rahier et al., this dispersion peak can be attributed to the amorphous aluminosilicate gel, which is the primary binder phase present in geopolymeric systems [26,27]. In addition, characteristic diffraction peaks of quartz remained after geopolymerization, which suggests that quartz did not participate in the geopolymerization reaction while the amorphous structure of metakaolin transformed from one structure to another structure. This is mainly due to the low dissolution of crystalline quartz into alkaline media [28,29]. Nevertheless, the solid-phase reaction between reserved quartz, undissolved amorphous silicon and aluminum structures in metakaolin with unreacted alkali activator might continue for a considerable time. This might be a reason that the compressive strength of geopolymer continually increases over time [30].

From the study of mechanical properties of alkali activated metakaolin-based geopolymer, the compressive strength of geopolymer increased with temperature during the initial 24 h of curing. The compressive strength of geopolymer reached over 40 MPa after 24 h curing at 60 °C, while it was only 11 MPa at 20 °C. However, the distinctions between the X-ray diffractograms of the geopolymers cured at different temperatures were not obvious. In addition, although the compressive strength of geopolymer cured for 168 h was obviously higher than if cured for 24 h at 60 °C, the differences in X-ray diffractograms were insignificant as well. It is suggested that the amorphous gel phase may be a major factor in the mechanical properties of alkali activated metakaolin-based geopolymers.

**Figure 3:**
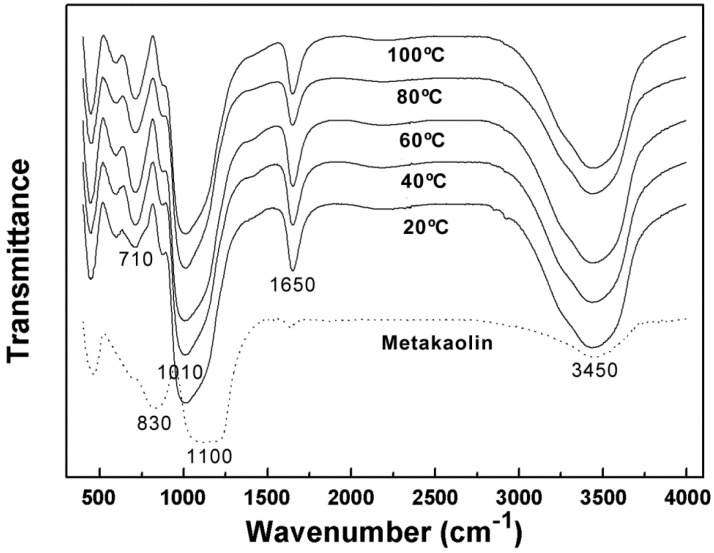
FT-IR spectra of metakaolin and geopolymers cured at different temperature for 24 h.

Figure 3 shows the FT-IR transmittance spectra of raw material metakaolin and the geopolymers cured at different temperatures for 24 h. Due to the presence of water in metakaolin and the geopolymers, the strong characteristic peaks approximately 3450 cm^−1^ and 1650 cm^−1^ were attributed to stretching and bending vibrations of hydroxyl, respectively.

After geopolymerization, the chemical environment around regular arranged chain structures of the Si-O bond altered, along with the formation of Al-O-Si bonds. Subsequently, the strong asymmetrical stretching vibration peak of the Si-O bond in metakaolin (1100 cm^−1^) all shifted to a lower wavenumber (to 1010 cm^−1^) for all the curing temperatures [31]. This indicated that the solidification process of the geopolymer is also a chemical reaction, with the generation of a new substance. In all the spectra of geopolymers, the bands at approximately 600 cm^−1^ were due to Al-O-Si stretching vibrations. Si-O-Si bending vibration at approximately 450 cm^−1^ was also present [32,33]. It was also found that the broad and strong peak at approximately 830 cm^−1^, which belongs to the stretching vibration of hexa-coordinate Al(VI)-OH and Al(VI)-O in metakaolin, almost disappeared after geopolymerization. A new peak at approximately 710 cm^−1^ from the bending vibration of tetra-coordinated Al(IV)-O-Si in a cyclic structure emerged on the FT-IR spectra of the geopolymers. This phenomenon signified the formation of aluminosilicate networks with the transition from hexa-coordinated Al(VI) to tetra-coordinated Al(IV) during the geopolymerization process, as observed by Sitarz et al. [34,35]. However, the transition was incomplete after 24 h of curing at 20 °C; therefore, the peak was not as sharp as in the higher curing temperature condition. In addition, after geopolymerization, all FT-IR spectra showed a shoulder at approximately 920 cm^−1^, due to Al(VI)-O residues, which proved that, after 24 h, the geopolymerization was incomplete, regardless of the curing temperature [36].

Table 5 lists the wavenumber and transmittance of the peaks at approximately 1010 cm^−1^ and 710 cm^−1^, before and after curing at different temperatures. A smaller value of transmittance represents a higher peak intensity. It was found that after curing both the shifting extent and peak intensity were highest on the alkali activated metakaolin-based geopolymer cured at 60 °C, although the difference was very slight. Therefore, it is speculated that the curing speed was fastest at 60 °C, which is consistent with the earlier study on the compressive strength of the geopolymers.

### 3.3. Alkali Activated Metakaolin-Based Geopolymer for Thermal Insulation

The traditional method for improving insulation in geopolymers is to introduce hole structures by physical or chemical methods, to produce geopolymers with high porosity and thus better thermal insulation. Aluminum powder, hydrogen peroxide, surfactant, polystyrene foam and high temperature are commonly used approaches. To further reduce the thermal conductivity of porous geopolymers, the bubbles should be more homogenous and closed. HMGBs are a novel filler for thermal insulation materials that contain vacuum rarefied gas inside glass beads and, unlike traditional foaming agents, feature light-weight, large volume, low thermal conductivity, controllable particle size distribution, high compressive strength, excellent dispersibility and fluidity, chemical inertness, and so on [37]. In this paper, HMGB K1 and HMGB VS-5500 were mixed with metakaolin particles to improve the thermal insulation properties of alkali activated metakaolin-based geopolymer based on different volume ratios with metakaolin. Excluding HMGB, the samples were set with the weight ratios referred to as sample #5 in Section 3.1.

**Figure 4:**
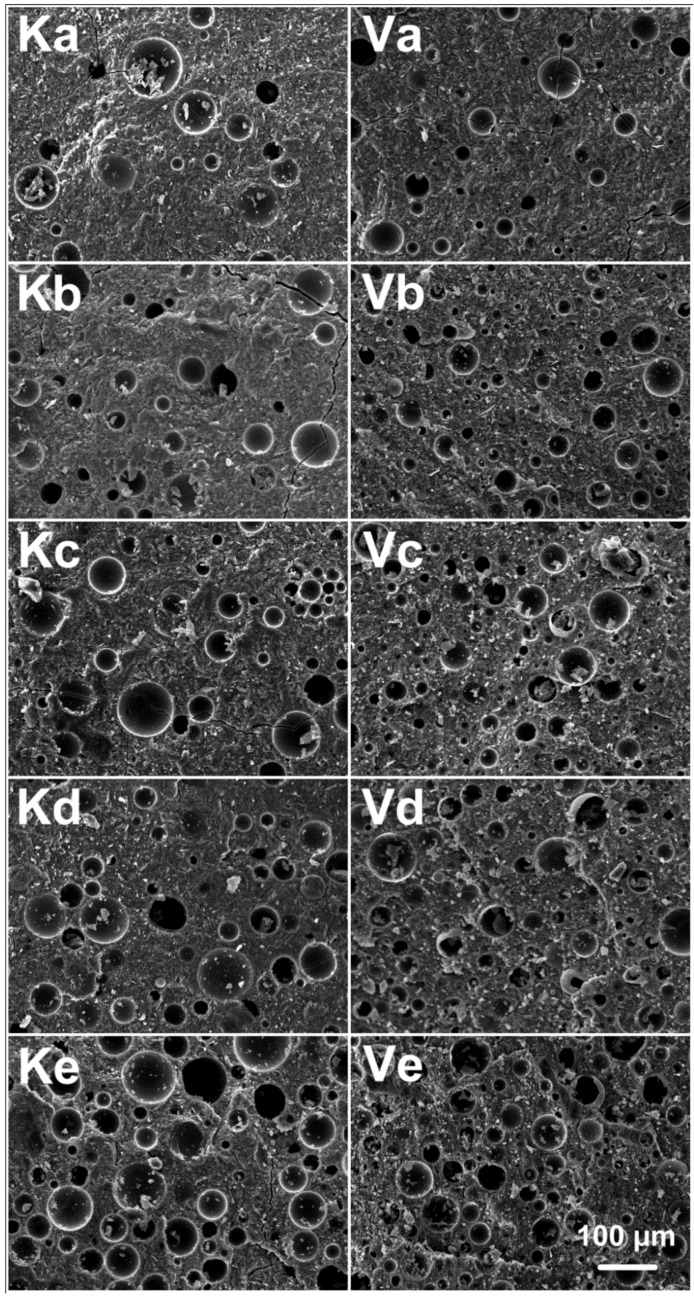
Internal morphologies of alkali activated metakaolin-based geopolymers with different ratios of HMGB: Ka–Ke denote the volume ratios of metakaolin to HMGB K1 of 10:1, 8:1, 6:1, 4:1 and 2:1, respectively; Va–Ve denote the volume ratios of metakaolin to HMGB VS-5500 of 10:1, 8:1, 6:1, 4:1 and 2:1, respectively. Scale bar indicates 100 μm.

As shown in Figure 4, the internal morphologies of alkali activated metakaolin-based geopolymers with different ratios of HMGB were studied by SEM after curing at 60 °C for 168 h. Images of Ka–Ke display the geopolymer with HMGB K1, with the volume ratio of metakaolin to HMGB K1 of 10:1, 8:1, 6:1, 4:1 and 2:1. Similarly, images of Va–Ve display the geopolymer with HMGB VS-5500 and the volume ratio of metakaolin to HMGB K1 of 10:1, 8:1, 6:1, 4:1 and 2:1. From the SEM images, we can see that the HMGB were distributed across the compact microstructures of the matrix of alkali activated metakaolin-based geopolymer randomly. An almost continuous matrix from the geopolymer paste to the glass beads can be observed, demonstrating a good interface between the two. However, some microcracks were observed in both HMGB samples. Such cracks could be reasonably imputed to the curing process, due to the constraining effect exerted by the glass spheres to the shrinkage of the paste [29]. With the increased admixing amount of HMGB, the distribution of sphere structures became denser and uniform. Since the average diameter of HMGB VS-5500 was smaller than that of HMGB K1, with a narrower size distribution, the spheres distributed more densely on the HMGB VS-5500 modified samples.

**Figure 5:**
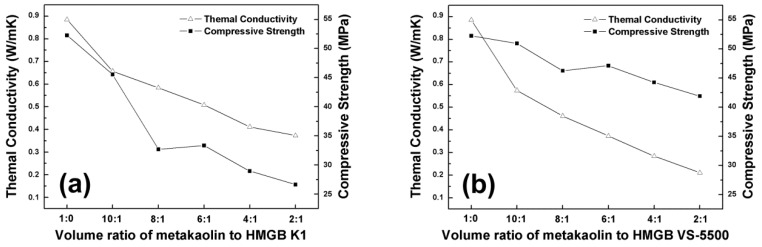
Thermal conductivity and compressive strength of alkali activated metakaolin-based geopolymers with different ratios of HMGB: (**a**) HMGB K1 and (**b**) HMGB VS-5500.

As the two important properties of thermal insulation materials, the thermal conductivity and compressive strength of alkali activated metakaolin-based geopolymers and HMGB modified geopolymers were tested after curing at 60 °C for 168 h [38]. As illustrated in Figure 5, the thermal conductivity and compressive strength of as-prepared alkali activated metakaolin-based geopolymers was 0.88 W/mK and 52.2 MPa, respectively. As the volume ratio of added metakaolin to HMGB K1 decreased from 10:1 to 2:1, the thermal conductivity reduced from 0.66 W/mK to 0.37 W/mK. However, the compressive strength also declined from 45 MPa (10:1) to 26 MPa (2:1). The decrease of conductivity of the geopolymer was achieved by the existence of air bubbles introduced by HMGB; the higher the HMGB content, the lower the thermal conductivity. However, the addition of HMGB K1 led to a dramatic decline in compressive strength of the geopolymeric system. The compressive strength of HMGB K1 is only 1.72 MPa. Similarly, as the volume ratio of metakaolin to HMGB VS-5500 decreased from 10:1 to 2:1, the thermal conductivity and compressive strength of alkali activated metakaolin-based geopolymer reduced from 0.57 W/mK and 51 MPa (10:1) to 0.21 W/mK and 42 MPa (2:1). Due to the high compressive strength of HMGB VS-5500 (37.9 MPa) compared to HMGB K1, the decline of compressive strength in alkali activated metakaolin-based geopolymer with HMGB VS-5500 was much less obvious. In addition, the average diameter and density of HMGB VS-5500 was smaller than that of HMGB K1 with a narrower size distribution. Therefore, as demonstrated by the SEM studies, the more extensively distributed spheres in the HMGB VS-5500 modified samples may be the reason why the geopolymer showed a lower thermal conductivity, in spite of their higher density compared to K1.

From Figure 5a,b, it was interesting to find that the compressive strength curve of both HMGB K1 and HMGB VS-5500 modified samples showed a small raised plateau, before declining to the 6:1 volume ratio of metakaolin to HMGB. This could be due to the unique internal structure and stress distribution around this volume ratio, but the specific causes of this phenomenon need further investigation. The above data indicate that better thermal insulation properties but lower compressive strength can be expected with the increased content of HMGB. Therefore, a proper volume ratio of metakaolin to HMGB should be determined, in order to balance thermal insulation effects and mechanical properties. Based upon the conditions studied in this paper, the 6:1 volume ratio of metakaolin to HMGB VS-55001 was the most suitable, with a thermal conductivity of 0.37 W/mK and compressive strength of 50 MPa.

**Figure 6:**
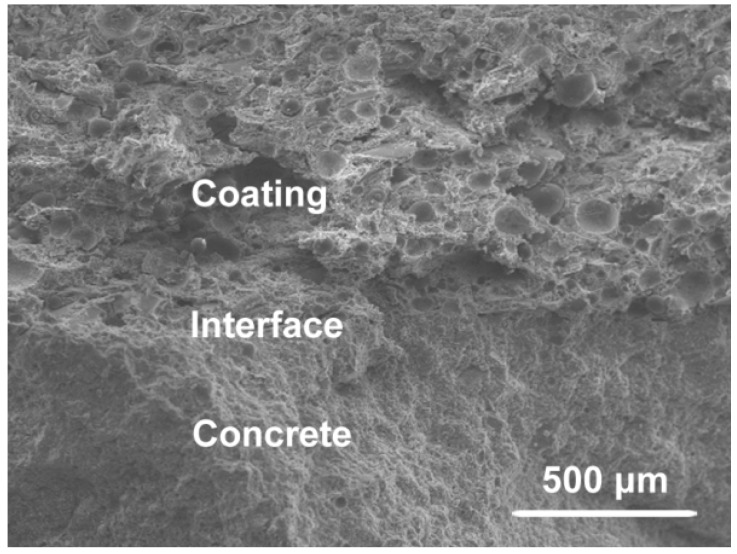
Cross section between geopolymer based thermal insulation coating and concrete. Scale bar indicated 500 μm.

One potential application of prepared glass bead modified geopolymer with low thermal conductivity is as a coating for concrete surface insulation protection. We explored the interfacial properties and durability of prepared glass bead modified geopolymer as a coating on concrete. The geopolymer containing HMGB VS-5500 was coated on a concrete surface with a thickness of 5 mm. After curing at 60 °C for 168 h, the cross section between the geopolymer based thermal insulation coating and concrete was studied by SEM. As illustrated in Figure 6, the coating adhered closely to the concrete, without any interspaces. Glass beads were also observed in the coating. In addition, the adhesive strength between the coating of geopolymer containing HMGB VS-5500 and concrete was 3.76 MPa. Artificial weathering aging tests were used to evaluate the durability of the geopolymer based thermal insulation coating. After 1000 cycles of irradiance and water spray alternation, the geopolymer showed excellent durability without any crushing or shelling. Although the curing condition was not feasible as it stands, it is believed that by adjusting the curing condition to milder conditions, as-prepared alkali activated metakaolin-based geopolymer could find widespread applications in concrete thermal protection.

## 4. Conclusions

In this paper, the preparation and properties of alkali activated metakaolin-based geopolymer were studied. It was found that the optimal geopolymer was prepared with metakaolin, sodium hydroxide, sodium silicate and water, using the weight ratios of 41.6%, 6.7% 45.0% and 6.7%, respectively. In addition, the best curing condition was found to be 60 °C for 168 h, where the alkali activated metakaolin-based geopolymer showed the highest compression strength at 52.26 MPa. The amorphous gel phase is the major factor in the mechanical properties of alkali activated metakaolin-based geopolymers. In addition, this result also suggests that a suitable volume ratio of metakaolin to hollow micro-sphere glass beads in alkali activated metakaolin-based geopolymers is 6:1, which achieved a thermal conductivity of 0.37 W/mK and compressive strength of 50 MPa.

## Figures and Tables

**Table 1:** Chemical composition (wt %) of metakaolin determined by X-ray Fluorescence (XRF).

SiO_2_	Al_2_O_3_	Fe_2_O_3_	CaO	K_2_O	Na_2_O	SO_3_	P_2_O_5_	TiO_2_	ZrO_2_
53.75%	43.82%	0.45%	0.16%	0.18%	0.26%	0.02%	0.43%	0.86%	0.029%

**Table 2:** Main performance parameters of HMGB K1 and HMGB VS-5500.

HMGB	Compressive Strength (MPa)	Density (g/cm^3^)	Size Distribution (μm, Volume Ratio)				Color
			10th%	50th%	90th%	Max	
K1	1.72	0.125	30	65	110	120	white
VS-5500	37.9	0.38	15	40	75	85	white

**Table 3:** Factor level of orthogonal experiment design.

Factor Level	Metakaolin (g)	NaOH (g)	Sodium Silicate (g)	Water (g)
1	24	3	25	4
2	25	4	26	5
3	26	5	27	6

**Table 4:** Orthogonal experiment of alkali activated metakaolin-based geopolymer with different ratios.

Sample	Metakaolin	NaOH	Sodium Silicate	Water	Compressive Strength	Standard Deviation
	(g)	(g)	(g)	(g)	(MPa)	(MPa)
#1	24.0	3.0	25.0	4.0	32.59	2.34
#2	24.0	4.0	26.0	5.0	30.24	3.69
#3	24.0	5.0	27.0	6.0	26.42	3.29
#4	25.0	3.0	26.0	6.0	34.40	3.59
#5	25.0	4.0	27.0	4.0	37.40	2.01
#6	25.0	5.0	25.0	5.0	29.32	3.72
#7	26.0	3.0	27.0	5.0	31.80	2.81
#8	26.0	4.0	25.0	6.0	32.43	3.77
#9	26.0	5.0	26.0	4.0	29.59	2.91
K_1_^a^	89.25	98.78	94.33	99.58		
K_2_	101.12	100.07	94.24	91.36		
K_3_	93.82	85.34	95.62	93.25		
k_1_^b^	29.75	32.93	31.44	33.19		
k_2_	33.71	33.36	31.41	30.45		
k_3_	31.27	28.45	31.87	31.08		
R ^c^	3.96	4.91	0.46	2.74		

^a^ K_1_ denoted the sum of level 1; ^b^ k_1_ denoted the average of level 1; ^c^ R denoted the range.

**Table 5:** Detailed data about the transmittance peak approximately 1010 cm^−1^ and 710 cm^−1^.

Curing Temperature (°C)	Transmittance Peak at Approximately 1010 cm^−1^		Transmittance Peak at Approximately 710 cm^−1^	
	Wavenumber (cm^−1^)	Transmittance	Wavenumber (cm^−1^)	Transmittance
None	1110	0.415	831	0.578
20	1018	0.053	715	0.796
40	1014	0.082	713	0.702
60	1008	0.045	709	0.656
80	1014	0.095	717	0.742
100	1012	0.059	715	0.702
